# Induced pluripotent stem cell-derived cells model brain microvascular endothelial cell glucose metabolism

**DOI:** 10.1186/s12987-022-00395-z

**Published:** 2022-12-09

**Authors:** Callie M. Weber, Bilal Moiz, Sophia M. Zic, Viviana Alpízar Vargas, Andrew Li, Alisa Morss Clyne

**Affiliations:** University of Maryland, College Park, MD 20742 USA

**Keywords:** Induced pluripotent stem cells, Brain microvascular endothelial cells, Glucose metabolism

## Abstract

Glucose transport from the blood into the brain is tightly regulated by brain microvascular endothelial cells (BMEC), which also use glucose as their primary energy source. To study how BMEC glucose transport contributes to cerebral glucose hypometabolism in diseases such as Alzheimer’s disease, it is essential to understand how these cells metabolize glucose. Human primary BMEC (hpBMEC) can be used for BMEC metabolism studies; however, they have poor barrier function and may not recapitulate in vivo BMEC function. iPSC-derived BMEC-like cells (hiBMEC) are readily available and have good barrier function but may have an underlying epithelial signature. In this study, we examined differences between hpBMEC and hiBMEC glucose metabolism using a combination of dynamic metabolic measurements, metabolic mass spectrometry, RNA sequencing, and Western blots. hiBMEC had decreased glycolytic flux relative to hpBMEC, and the overall metabolomes and metabolic enzyme levels were different between the two cell types. However, hpBMEC and hiBMEC had similar glucose metabolism, including nearly identical glucose labeled fractions of glycolytic and TCA cycle metabolites. Treatment with astrocyte conditioned media and high glucose increased glycolysis in both hpBMEC and hiBMEC, though hpBMEC decreased glycolysis in response to fluvastatin while hiBMEC did not. Together, these results suggest that hiBMEC can be used to model cerebral vascular glucose metabolism, which expands their use beyond barrier models.

**Supplementary Information:**

The online version contains supplementary material available at 10.1186/s12987-022-00395-z.

## Introduction

Glucose is the primary energy source in the brain [1, 2], and brain glucose hypometabolism has been implicated in neurodegenerative diseases such as Alzheimer’s disease [3–8]. Glucose transport into the brain is dynamically regulated by brain microvascular endothelial cells (BMEC), which together with pericytes and astrocytes form the neurovascular unit (NVU). BMEC have extensive intercellular tight junctions that block paracellular transport of metabolites such as glucose into the brain, allowing diffusion of only lipophilic compounds with a molecular weight less than 400 Da [9] and less than 8 hydrogen bonds with water [10]. Instead, glucose is transported into the brain transcellularly via glucose transporters such as GLUT1.

Peripheral endothelial cells are thought to rely on glycolysis rather than oxidative phosphorylation for ATP production [11, 12], and as such have a small mitochondrial volume [13]. Elevated glycolytic rates reduce peripheral endothelial cell reliance on oxygen, which may both reduce metabolic stress and enhance lactate signaling as endothelial cells undergo angiogenesis into a hypoxic environment [14–16]. In contrast, relatively little is known about BMEC metabolism. BMEC likely also use glucose as their primary energy source. However, BMEC may have a higher metabolic flexibility, since they maintained viability in the absence of glucose [17], have higher mitochondrial volume than peripheral EC [18], and switched to oxidative phosphorylation of glutamate in hypoglycemia [19]. It is therefore essential to understand how glucose that is taken up by BMEC is metabolized versus transported to the NVU and the rest of the brain.

In vitro blood–brain barrier (BBB) models are an important tool for understanding glucose metabolism and transport since in vivo systems are complex and difficult to analyze. Currently, the BBB can be modeled in vitro using human primary BMEC (hpBMEC), the immortalized hCMEC/D3 cell line, or induced pluripotent stem cell derived BMEC-like cells (hiBMEC). hpBMEC are expensive and difficult to obtain, as their isolation is complicated by low yield, mural cell contamination, and ethical issues. hpBMEC are also prone to donor variability and low barrier integrity [20]. hCMEC/D3 cells were developed to overcome some of the hpBMEC limitations; however, these cells also have low barrier integrity and tight junction protein expression [21]. Previous evaluations of hCMEC/D3 cells show they have similar glycolytic and oxidative metabolism to hpBMEC [22]. hiBMEC offer a renewable BMEC source with high barrier integrity and tight junction protein expression [23]. However, recent studies suggest that these cells retain an epithelial-like transcriptomic profile and may therefore not fully recapture the vascular BMEC phenotype [24, 25].

Little is currently known about glucose metabolism in any of the in vitro human BMEC cell lines, and therefore selecting a cell type to use in glucose metabolism and transport studies is challenging. In this study, we compared glucose metabolism between hpBMEC and hiBMEC. We used transcriptomic and metabolomic profiling, together with real-time metabolic analysis, to analyze glucose metabolism in hpBMEC and hiBMEC at baseline and when treated with stimuli known to alter endothelial glucose metabolism. Our data show key differences in baseline hpBMEC and hiBMEC glucose metabolic flux and metabolic enzyme expression; however, both cell types had similar baseline glucose metabolomic profiles. We further show that hpBMEC and hiBMEC respond similarly to most of our tested metabolic stimuli. Together, our data suggest that hpBMEC and hiBMEC provide similar results in an in vitro model of BMEC glucose metabolism.

## Methods

### hiBMEC differentiation

hiBMEC were differentiated using previously established protocols [23]. IMR90 iPSC [26], a generous gift from Dr. Xiaoming He, were maintained in mTeSR-Plus medium (STEMCELL Technologies, 100–0276) on Matrigel (Corning, 354230) and passaged using Versene (Thermo Fisher, 15040066) at 70% confluence. For differentiation, cells at 70% confluence were detached using Accutase (Thermo Fisher, A1110501) and seeded at 150,000 cells/well on Matrigel-coated plates in mTESR-Plus medium containing 10 µM Y-27632 (ROCK inhibitor; Tocris, 1254). Over the subsequent 4 days, medium was changed to E6 (STEMCELL Technologies, 05946) and replaced daily. On day 4, cells were changed to either human endothelial serum free media (hESFM; Thermo Fisher, 11111044) or neurobasal medium (Thermo Fisher, 21103049) supplemented with 2% B27 (Thermo Fisher, 17504001), 20 ng/mL basic fibroblast growth factor (bFGF; Peprotech, 100-18B), and 10 µM retinoic acid (RA; Millipore Sigma, R2625-50MG). Media was not changed on day 5. On day 6, the cells were subcultured at 1 × 10^6^ cells/cm^2^ onto extracellular matrix (ECM) containing 0.4 mg/mL collagen IV (Millipore Sigma, C7521) and 0.1 mg/mL fibronectin (Millipore Sigma, F2006) on 0.4 μm pore Transwell filters (Corning, 3460) or 12-well plates (CELLTREAT, 229112).

### hpBMEC culture

hpBMEC were purchased from Cell Systems (ACBRI 376 V) and only used up to passage 9, since higher passages are associated with decreased proliferation which could alter cell metabolism [27, 28]. Cells were maintained in endothelial cell growth medium-2 microvascular (EGM-2 MV; Lonza, CC-4147) supplemented with 1% glutamine (Thermo Fisher, 25–030-081), 1% penicillin streptomycin (Thermo Fisher, 15140163), and 10% fetal bovine serum (FBS; Cytiva, SH30088.03). For experiments, cells were seeded on 0.4 μm pore Transwell filters or 12-well plates coated with ECM as previously described.

### Astrocyte culture

Primary human astrocytes were a generous gift from Dr. Silvia Muro and used through passage 9. Astrocytes were maintained in Dulbecco’s Modified Eagle Medium (DMEM; Thermo Fisher, A1443001) supplemented with 5.5 mM glucose, 15% FBS, 1% penicillin streptomycin, and 1% glutamine. Conditioned media was collected from confluent astrocytes after 24 h, aliquoted, and frozen until use.

### TEER

To measure transendothelial electrical resistance (TEER), BMEC were subcultured onto 0.4 μm ECM-coated Transwell filters in hESFM with 2% B27, 20 ng/mL bFGF, and 10 µM RA. Every day post-subculture, TEER was measured in triplicate using STX2-Plus electrodes and the Epithelial Volt/Ohm Meter 3 (EVOM3; World Precision Instruments).

### YSI

**Fig. 1:**
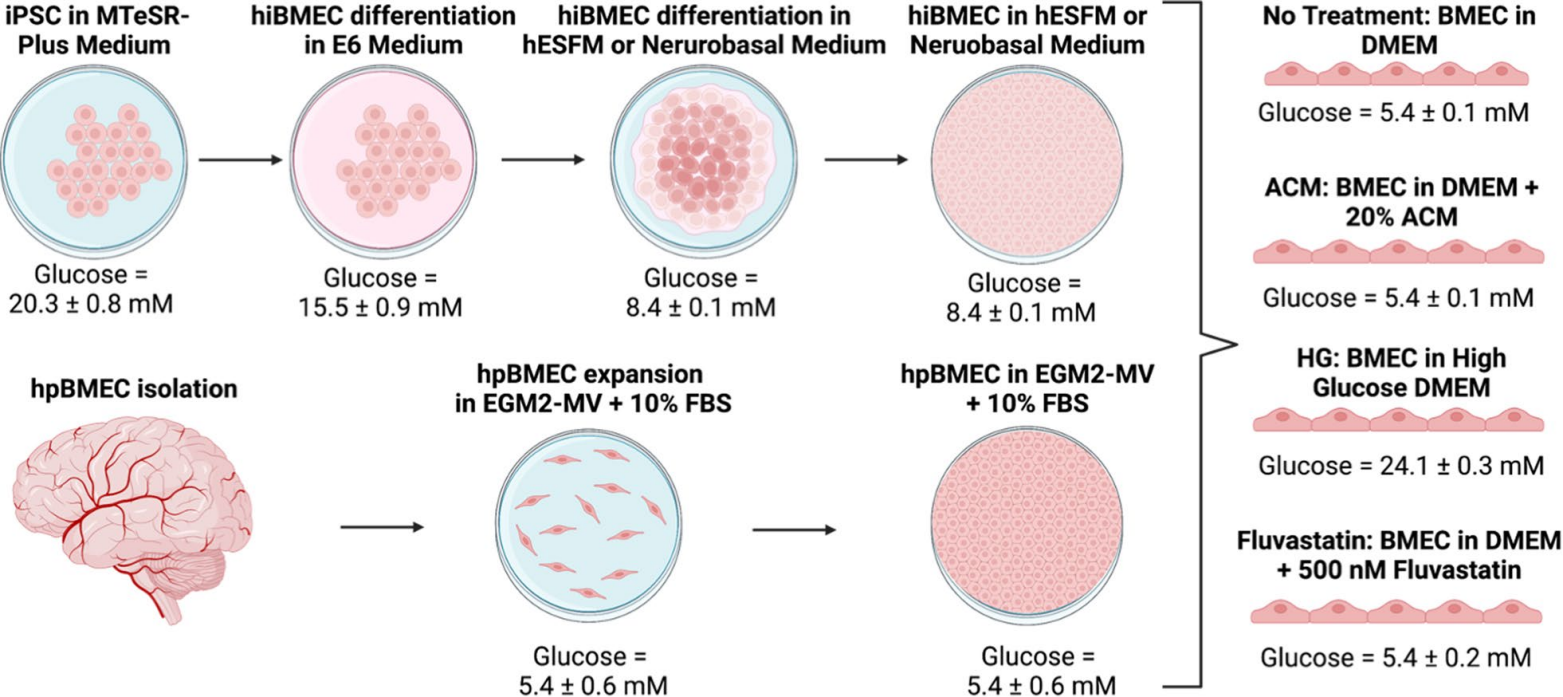
Glucose concentrations in cell culture conditions. Glucose concentrations were measured via YSI at each step of the hiBMEC differentiation process, in hpBMEC culture, and in each treatment media. Concentrations are reported as mean ± standard deviation. n = 12 biological replicates

Media metabolite concentrations were measured using a YSI 2950 Bioanalyzer (Yellow Springs Instruments, 527690). BMEC were subcultured onto 12-well plates in hESFM with 2% B27, 20 ng/mL bFGF, and 10 µM RA. The next day, media was changed to DMEM supplemented with 4.5 mM glutamine, 2% B27, and either 5.5 mM glucose (no treatment), 25 mM glucose, 20% astrocyte conditioned media or 500 nM fluvastatin (Millipore Sigma, SML0038-10MG; Fig. 1). 200 μL media samples were taken from each well of the 12-well plates. Glucose and lactate concentrations were then quantified using the YSI.

### Seahorse assays

BMEC glycolysis was measured using the Seahorse Glycolytic Rate Assay (Agilent, 103344–100) and oxidative respiration was measured using the Seahorse Mito Stress Test (Agilent, 103015–100). hiBMEC were subcultured at 100,000 cells/well in a 96-well Seahorse Assay plate in hESFM supplemented with 2% B27, 20 ng/mL bFGF, and 10 µM RA. The next day, media was replaced with hESFM supplemented with only B27. hpBMEC were seeded in the Seahorse Assay plate at 10,000 cells/well in EGM-2 MV and cultured for 48 h. Three days after seeding, all cells were switched to DMEM supplemented with 5.5 mM glucose, 4.5 mM glutamine, and 2% B27. On the day of the assay, media was changed to Seahorse DMEM (Agilent, 103680–100) supplemented with 5.5 mM glucose, 1 mM pyruvate, and 4.5 mM glutamine for 1 h. For the Glycolytic Rate Assay, rotenone + antimycin A (0.5 µM) and 2 deoxyglucose (50 mM) were added to the drug loading cartridge. For the Mito Stress Test, oligomycin (1.5 µM), FCCP (0.5 µM), and rotenone + antimycin A (0.5 µM) were added to the drug loading cartridge. Glycolytic proton efflux rate (GlycoPER, glycolysis) and oxygen consumption rate (OCR, oxidative respiration) were measured using a Seahorse XF96 (Agilent) and analyzed using Seahorse analytics software. To normalize rates to cell count, Seahorse plates were fixed with 4% PFA, stained with Hoescht (Thermo Fisher 62249, 1:2000), washed twice with PBS, and imaged using an Eclipse Ti2 spinning disk confocal microscope (Nikon) with a 10X objective.

### Immunofluorescence microscopy

For immunofluorescence, BMEC were subcultured on glass coverslips. Cells were fixed in 4% paraformaldehyde (PFA; Millipore Sigma, P6148) for 20 min, after which they were blocked and permeabilized in 5% normal goat serum (Millipore Sigma, S26) with 0.2% TritonX-100 (Alfa Aesar, A16046) in phosphate buffered saline (PBS; Thermo Fisher, 70011069) for 1 h. Cells were incubated in primary antibodies for GLUT-1 (Thermo Fisher, 21829–1-AP, 1:100), zona occludins-1 (ZO-1; Cell Signaling Technology, 13663S, 1:100), and occludin (Cell Signaling Technology, 91131S, 1:100) overnight at 4°C. Cells were then incubated in a goat-anti-rabbit secondary antibody (Thermo Fisher, A-11012, 1:1000) with Hoechst (Thermo Fisher, 62249, 1:2000) for 1 h. Samples were imaged using an Eclipse Ti2 spinning disk confocal microscope (Nikon) with a 60× oil objective.

### RNA-Sequencing

hiBMEC RNA was isolated using a phenol/chloroform isolation protocol [29] and quantified using a Nanodrop 2000c (Thermo Fisher). RNA was sequenced at Novogene (Sacramento, CA) using a NovaSeq 6000 with 105-bp paired end reads. Reads were trimmed and mapped to the human genome and converted to fragments per kilobase of transcript per million mapped reads (FPKM) using Galaxy open-source software. RNA sequencing data from hpBMEC was accessed using publicly available data [24, 30, 31].

### Western blots

hiBMEC and hpBMEC were grown to confluence in their respective media and then changed to DMEM supplemented with 5.5 mM glucose, 4.5 mM glutamine, and 2% B27 for 24 h. After treatment, cells were lysed in RIPA buffer (Thermo Fisher, 89901) supplemented with Halt Protease and Phosphatase Inhibitor (Fisher Scientific, PI78440), and protein was quantified via BCA assay (Thermo Fisher, 23225). 3.5 µg/µL protein, 7.5 µL sample buffer (Thermo Fisher, NP0008) and 3 µL reducing agent (Thermo Fisher, NP0009) were combined, heated to 37°C (GLUT1) or 70°C (all other proteins) for 5 min, then loaded in 4–12% Bis–Tris gels (Thermo Fisher; NP0323). Samples were transferred to nitrocellulose or polyvinylidene fluoride membranes (Thermo Fisher, IB23001 and IB24001) using an iBlot2 (Thermo Fisher, IBL21001), blocked for 1 h in 5% bovine serum albumin (Millipore Sigma, 126609) in PBS containing 0.5% Tween 20 (Thermo Fisher, 85,113), and incubated in primary antibodies (Table 1) overnight. The next day, membranes were incubated for 2 h in the respective secondary antibody (Table 1) and imaged using an Alpha Innotech Fluorchem Imager (Protein Simple). Band intensities were analyzed using AlphaView software.

**Table 1:** Western blot antibodies

Antibody Name	Company	Catalog Number
GLUT1	Thermo Fisher	21829–1-AP
HXK I	Santa Cruz Biotechnology	sc-46695
HXK II	Santa Cruz Biotechnology	sc-374091
GPI	Santa Cruz Biotechnology	sc-365066
PFKFB3	Cell Signaling Technology	13123S
PFK1	Santa Cruz Biotechnology	sc-166722
AldoA	Santa Cruz Biotechnology	sc-390733
GAPDH	Cell Signaling Technology	2118S
PGK	Santa Cruz Biotechnology	sc-48342
PGM-1	Santa Cruz Biotechnology	sc-373796
α-Enolase	Santa Cruz Biotechnology	sc-100812
PKM	Santa Cruz Biotechnology	sc-365684
LDH	Santa Cruz Biotechnology	sc-133123
CPT1A	Cell Signaling Technology	12252S
CPT1B	ABCAM	ab134135
Citrate Synthetase	ABCAM	ab96600
IDH1	Cell Signaling Technology	8137S
IDH2	Millipore Sigma	HPA007831
ß-Actin	Santa Cruz Biotechnology	sc-47778
Anti-mouse IgG (H + L), HRP Conjugate	Promega	W4021
Anti-rabbit IgG (H + L), HRP Conjugate	Promega	W4011

### Metabolic mass spectrometry

Both hiBMEC and hpBMEC were cultured for 24 h in serum-free DMEM (Thermo Fisher, A1443001) supplemented with 2% B27, 200 mM L-glutamine, and 5.5 mM U-^13^C_6_-glucose (Fisher Scientific, NC9207695). The medium was then removed, and 80:20 methanol:water (extraction solvent) was added to cells for 15 min at −80°C. Cells were scraped in the extraction solvent, vortexed, and then centrifuged at 16,000 g for 10 min at 4°C to pellet debris. Cell metabolite extracts were then shipped to the University of Colorado School of Medicine Metabolomics Core. Samples were randomized and 8 µL was injected onto a Q Exactive mass spectrometer (MS) by a Vanquish ultra-high performance liquid chromatograph (UHPLC; ThermoFisher, San Jose, CA, USA) as described previously [32]. Electrospray ionization was used to introduce eluent to the MS, which scanned in full MS mode (2 µscans) over 65–950 m/z. Technical mixes were injected approximately every ten samples to determine instrument stability [33]. Metabolites were manually annotated and integrated with Maven (Princeton University) in conjunction with the KEGG database. Peak quality was determined using blanks, technical mixes, and ^13^C natural abundance [34]. Isotope labeling was corrected using the IsoCor Python package [35]. Metabolite pool size was analyzed using Metaboanalyst 5.0 [36].

### Statistics

Statistics were analyzed in GraphPad Prism. Non-parametric Mann–Whitney tests were used to compare datasets, and data were considered biologically significant if p < 0.05 and Log_2_FC > 0.6. RNA-sequencing fold change was considered biologically significant with Log_2_FC > 0.6. Significance of metabolomics data was corrected using the Benjamini–Hochberg correction and was considered biologically significant if Log_2_FC > 2.32.

## Results

hiBMEC are increasingly used as a human in vitro BBB model for drug delivery and disease modeling [37, 38]. As brain glucose hypometabolism is a common indicator of neurodegenerative diseases, we are interested in using hiBMEC to study glucose transport across the BBB. Since glucose is metabolized as well as transported by BMEC, in this study we compared hiBMEC and hpBMEC glucose metabolism.

### hiBMEC have higher barrier function but lower glycolytic rate relative to hpBMEC

**Fig. 2:**
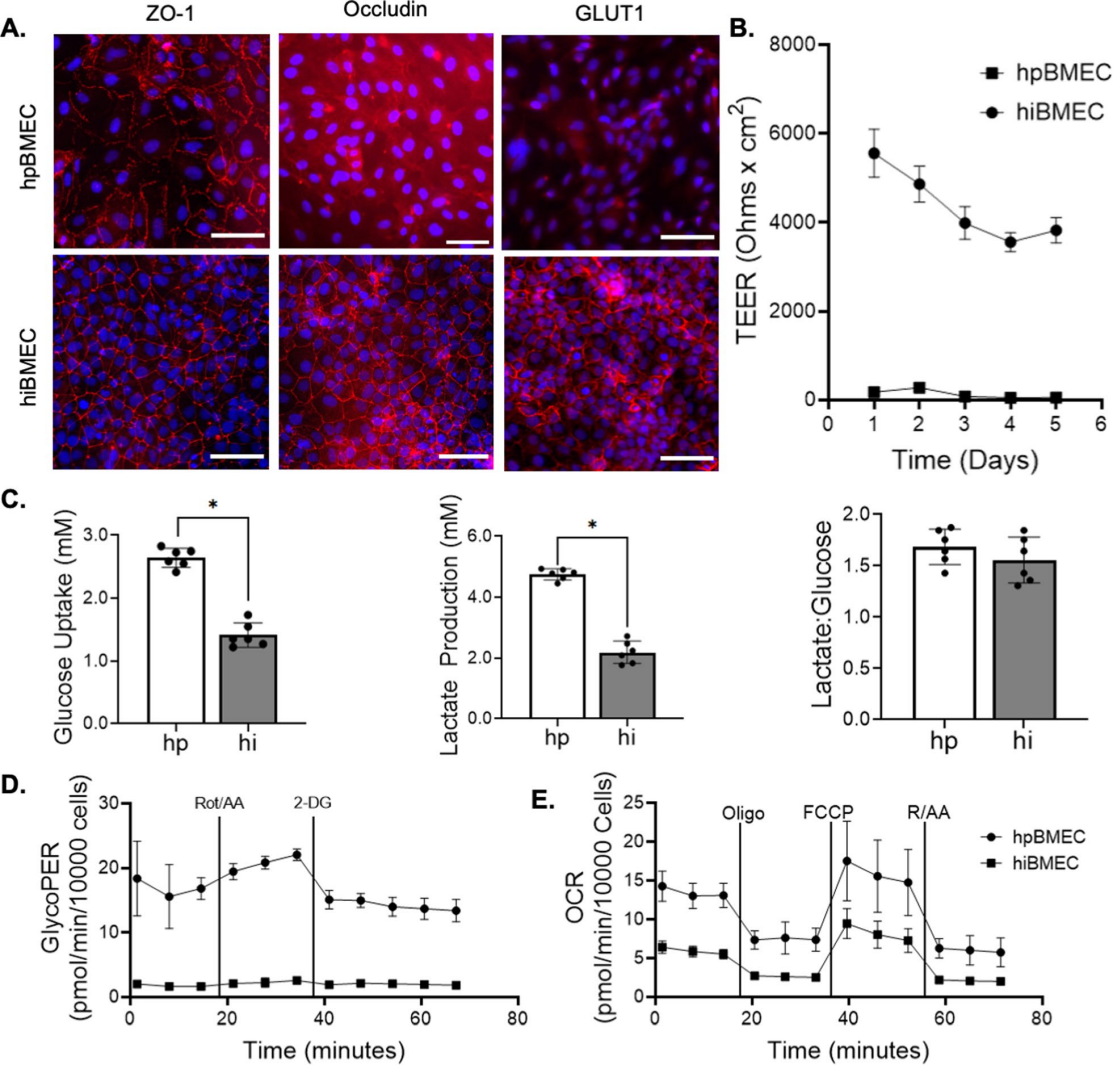
hpBMEC had higher glycolytic and oxidative glucose metabolism than hiBMEC, but similar proportions of glycolytic glucose metabolism. **A** Representative confocal images of GLUT1, ZO-1, occludin (all red), and hoechst (nuclei, blue) in hpBMEC and hiBMEC. Scale = 100 µm. **B** TEER of hpBMEC and hiBMEC measured on confluent monolayers on 0.4 µm pore Transwell filters. **C** Glucose uptake, lactate production, and lactate:glucose ratio in hpBMEC and hiBMEC cultured in 5.5 mM glucose over 24 h, as measured by YSI. **D** GlycoPER (glycolysis) and **E **OCR (oxidative respiration) in hpBMEC vs. hiBMEC measured via Seahorse assays, normalized to cell count. n = 6 biological replicates; *p < 0.05 using a Mann–Whitney

We first confirmed the glucose transporter and barrier proteins of hpBMEC and hiBMEC using immunofluorescent imaging for GLUT1 and tight junction proteins ZO-1 and occludin (Fig. 2A). TEER was then used to analyze hpBMEC and hiBMEC barrier function. hiBMEC had a higher maximum TEER (5555 Ω x cm^2^) compared to hpBMEC (283 Ω x cm^2^; p < 0.0001; Fig. 2B), which is similar to previously reported values [20, 23, 39–41].

We next measured BMEC glucose metabolism via glycolysis and oxidative respiration. hpBMEC took up more glucose (2.64 mM vs. 1.41 mM; p = 0.002) and produced more lactate (4.75 mM vs. 2.19 mM; p = 0.002) compared to hiBMEC (Fig. 2C). However, the lactate:glucose ratio (~1.6 ± 0.2) was similar between hpBMEC and hiBMEC, indicating that in both cell types, a similar proportion of glucose was metabolized via glycolysis. Similarly, hpBMEC demonstrated higher basal GlycoPER (glycolysis; 16.80 vs. 1.65 pmol/min/1000 cells; p < 0.0001) and OCR (oxidative respiration; 13.07 vs. 5.52 pmol/min/1000 cells; p < 0.0001) compared to hiBMEC (Fig. 2D, E). Both cell types responded similarly to metabolic manipulation, including oligomycin (inhibits mitochondrial ATP synthase), FCCP (depolarizes mitochondrial membrane potential to maximize mitochondrial respiration), and rotenone/antimycin (shuts down mitochondrial respiration). Both cell types also produced more ATP from glycolysis than from oxidative respiration (Additional file 1: Fig. S1 A, B). However, hpBMEC had higher glycolytic capacity (366%; p < 0.0001), maximal respiration (363%; p < 0.0001), proton leak (505%; p < 0.0001), spare capacity (528%; p < 0.0001), and ATP-linked respiration than hiBMEC (243%; p < 0.0001; Additional file 1: Fig. S1 C-G).

### hpBMEC and hiBMEC metabolomic profiles differ primarily in acylcarnetines

**Fig. 3:**
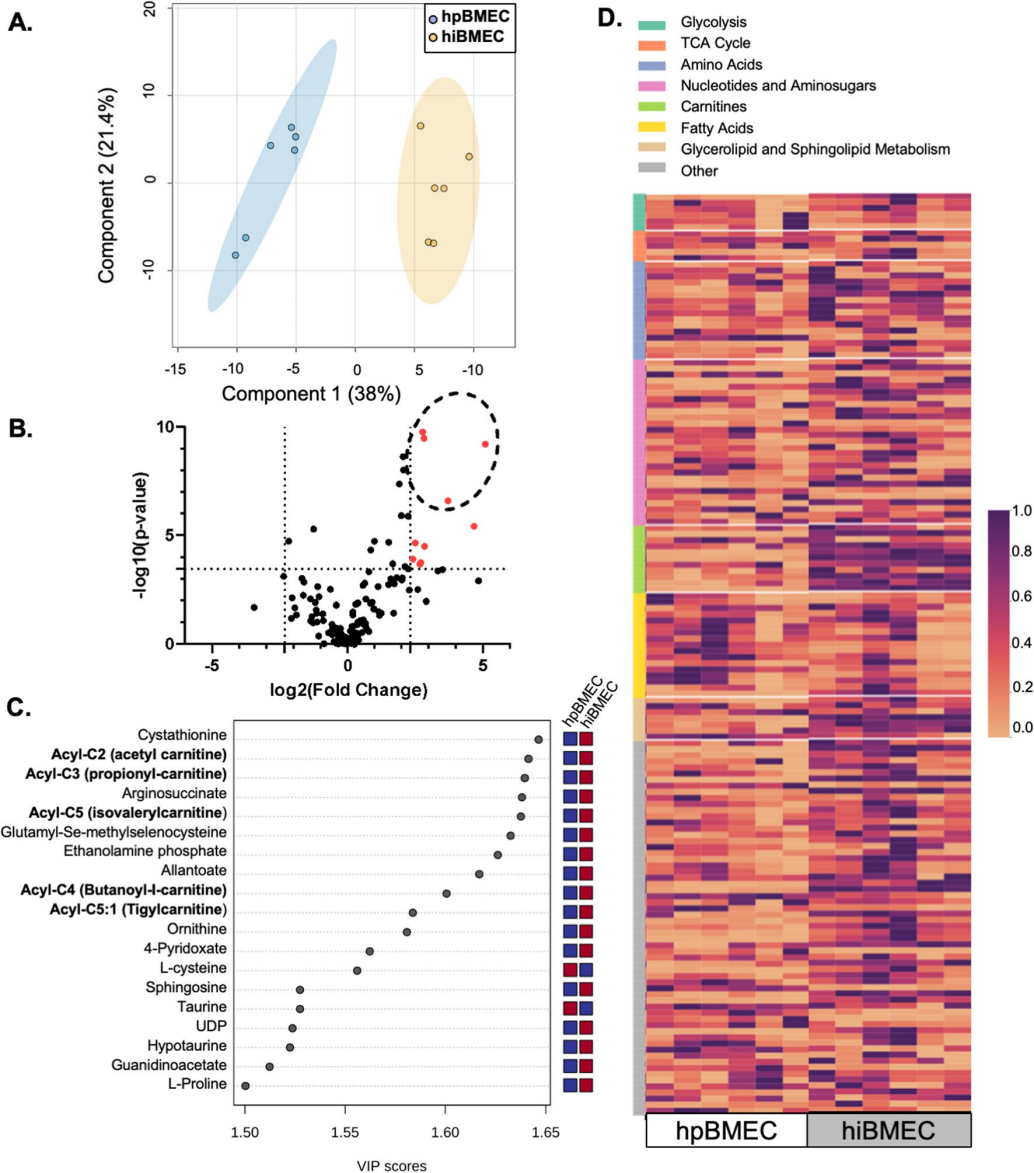
Metabolomic abundance profiling showed hpBMEC and hiBMEC have distinct metabolomes, largely driven by changed in acyl-carnitines. **A** Partial least squares discriminant analysis (PLS-DA) of metabolic LC–MS data from hpBMEC and hiBMEC cultured with 5.5 mM U-^13^C_6_-glucose for 24 h. **B** Log_2_(fold change) versus -log_10_(p-value), identifying metabolites that are statistically significantly different following Bonferroni correction (p < 0.0003) and biologically relevant (fold change > 2.32). Acyl-carnitines are circled. Plot generated in MetaboAnalyst 5.0. **C** Variable importance in projection (VIP) scores plot, showing metabolites with VIP scores greater than 1.50. Red box = higher and blue box = lower in each cell type. Generated in MetaboAnalyst 5.0. **D** Metabolomic heat map, organized by metabolomic subsystem. n = 6 biological replicates

To analyze global similarities and differences in hpBMEC and hiBMEC intracellular metabolites, we labeled cells with uniformly labeled glucose and then examined metabolite pool sizes as well as fractional enrichment patterns with LC–MS. We first used principal component analysis (PCA) to determine whether the hpBMEC and hiBMEC total metabolite pools segregated independently of labeling. hpBMEC and hiBMEC metabolomes separated along both components (Fig. 3A, PC1 = 38%, PC2 = 21.4%), indicating key metabolomic differences between the two cell types. Of the 150 metabolites detected, 12 were statistically significantly different and the differences were of biologically relevant scale (Log_2_FC > 2.32) between hpBMEC and hiBMEC (Fig. 3B). Two of these metabolites, argininosuccinate (Log_2_FC = 19.98 and p < 0.0001) and cystathione (Log_2_FC = 17.64 and p < 0.0001), were detected in hiBMEC but not in hpBMEC. Of the remaining metabolites, there were four acylcarnitines, two representatives from nucleotide metabolism, two from glutathione homeostasis, and one each from arginine/proline metabolism and glycolysis. The sole glycolytic metabolite was a phosphohexose glycolytic intermediate (such as glucose-6-phosphate, glucose-1-phosphate, and/or fructose-6-phosphate); however, its exact identity could not be deciphered due to overlap of signal among these compounds.

Partial-least squares discriminant analysis (PLS-DA), a supervised technique that maximizes variance between classes, was then used to identify variables that drove the glucose metabolic differences between hpBMEC and hiBMEC. Nineteen metabolites had variable importance in projection (VIP) scores greater than 1.5 (Fig. 3C); however, none of these metabolites is primarily involved in glycolytic, glycolytic side branch, or TCA cycle pathways. Finally, we used heat maps and hypergeometric analysis to identify statistically significant changes in metabolic pathways (Fig. 3D, Additional file 1: Table S1). Metabolite pool sizes were overall similar between the cell types. Twelve metabolic pathways had at least one metabolite that was statistically different between the hpBMEC and hiBMEC; however, we found statistically significant changes only in acylcarnitine (p = 0.014) and urea cycle (p = 0.0245) pathways.

### Glycolytic enzymes, but not labeled metabolite fraction, differ between hpBMEC and hiBMEC

**Fig. 4:**
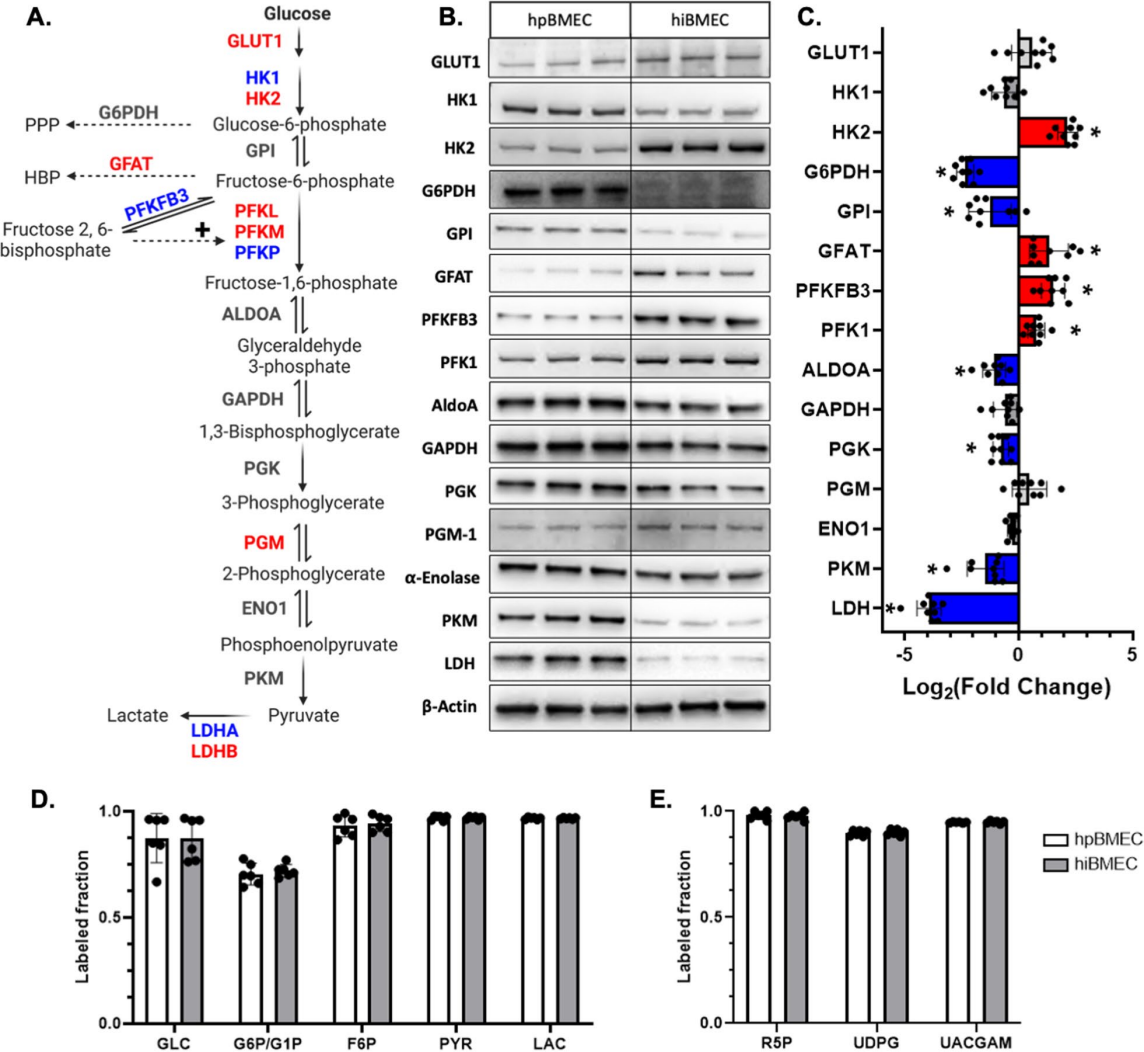
hpBMEC and hiBMEC showed differences in glycolytic enzyme gene and protein levels, but similar U-^13^C_6_-glucose labeled metabolite fractions. **A** Glucose transporter, glycolysis, and glycolysis side branch pathway enzyme gene expression, measured by RNA sequencing. Red = higher in hiBMEC, blue = higher in hpBMEC (LogFC > 0.6). n = 3 biological replicates. **B**, **C** Western blots with quantification of glucose transporter and glycolytic enzyme protein levels from hpBMEC and hiBMEC. Red = higher in hiBMEC, blue = higher in hpBMEC (significant with p < 0.05, LogFC > 0.6). Blots shown are representative of three separate experiments. n = 9 biological replicates. **D**, **E** Glucose labeled fraction of glycolytic (**D**) and glycolytic side branch pathway (**E**) metabolites in hpBMEC and hiBMEC incubated with U-^13^C_6_-glucose for 24 h and then analyzed by LC–MS. n = 6 biological replicates. *p < 0.05 and LogFC > 0.6 using a Mann–Whitney test

Since metabolism is partially driven by glucose transporter and glycolytic enzyme levels, we used RNA sequencing and Western blots to determine differences between hpBMEC and hiBMEC. Glucose transporter GLUT1 was upregulated in hiBMEC compared to hpBMEC (Log_2_FC > 0.6; Fig. 4A). Glycolytic enzymes hexokinase 2 (HK2), glutamine-fructose-6-phosphate transaminase (GFAT), phosphofructokinase, liver (PFKL), phosphofructokinase, muscle (PFKM), phosphoglucomutase (PGM), and lactate dehydrogenase B (LDHB) were also upregulated in the hiBMEC compared to hpBMEC (Log_2_FC > 0.6). However, hexokinase 1 (HK1), 6-phosphofructo-2-kinase/fructose-2,6-biphosphatase 3 (PFKFB3), phosphofructokinase, platelet (PFKP), and lactate dehydrogenase A (LDHA) were downregulated in hiBMEC compared to hpBMEC (Log_2_FC > 0.6; Fig. 4A). All other enzymes were similar for hiBMEC and hpBMEC. Protein levels overall agreed with the RNA data, although protein changes for G6PDH, GPI, ALDOA, PGK, and PKM reached statistical significance (p < 0.05 and Log_2_FC > 0.6; Fig. 4B, C) while RNA changes did not. GLUT1, HK1 and PGM, which were statistically significantly different in the RNA data, did not significantly change in the protein analysis. Western blots for PFK1 and LDH used non-isoform-specific antibodies and showed that despite differences in RNA expression for the different isoforms, PFK1 protein overall was higher (p = 0.0012) while LDH protein overall was lower in hiBMEC compared to hpBMEC (p < 0.0001). Only PFKFB3 disagreed between the RNA and protein data, with PFKFB3 RNA lower in hiBMEC (Log_2_FC = −1.37) and PFKFB3 protein higher in hiBMEC (Log_2_FC = 1.51; p < 0.0001). Overall, glycolytic enzyme RNA and protein levels showed mixed differences between hiBMEC and hpBMEC. Isotope labeling with U-^13^C_6_-glucose revealed no significant differences in the labeled enrichment of intracellular glycolytic (Fig. 4D) or glycolytic side branch pathway metabolites (Fig. 4E) in hpBMEC and hiBMEC.

### Mitochondrial metabolic enzymes, but not labeled metabolite fractions, differ between hpBMEC and hiBMEC

**Fig. 5:**
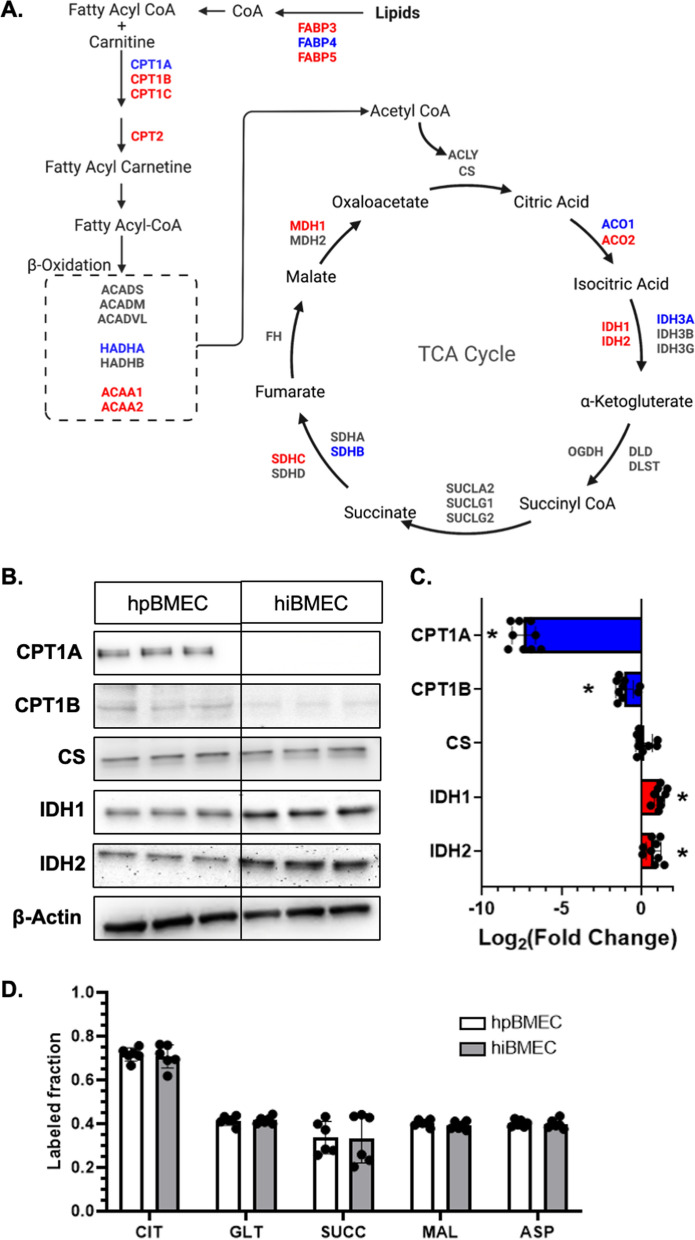
hpBMEC and hiBMEC had different TCA and FAO gene and protein levels, but similar U-^13^C_6_-glucose labeled metabolite fractions. **A** TCA cycle and FAO enzyme expression, measured by RNA sequencing. Red = higher in hiBMEC, blue = higher in hpBMEC (LogFC > 0.6). n = 3 biological replicates. **B**, **C** TCA cycle and FAO enzyme protein levels, measured by Western blot. Red = higher in hiBMEC, blue = higher in hpBMEC (significant with p < 0.05, LogFC > 0.6). Blots shown are representative of three separate experiments. n = 9 biological replicates. **D** Glucose labeled fraction of TCA metabolites in hpBMEC and hiBMEC incubated with U-^13^C_6_-glucose for 24 h and then analyzed by LC–MS. n = 6 biological replicates. *p < 0.05 and LogFC > 0.6 using a Mann–Whitney test

Metabolite pool size data suggested that hpBMEC and hiBMEC had large differences in acyl carnitines, which are important in mitochondrial metabolism. We therefore examined enzymes involved in mitochondrial metabolism, specifically the TCA cycle and fatty acid oxidation (FAO), by RNA sequencing and Western blot. From the RNA sequencing, 12 enzymes associated with the TCA cycle and FAO were upregulated and 6 were downregulated in hiBMEC compared to hpBMEC (Fig. 5A). Two isoforms of the rate-limiting TCA cycle enzyme, isocitrate dehydrogenase 1 and 2 (IDH1, IDH2) were upregulated in hiBMEC as compared to hpBMEC (Log_2_FC = 1.66 for IDH1; Log_2_FC = 0.67 for IDH1), while IDH3A was downregulated (Log_2_FC = −0.84). Carnitine palmitoyltransferase 1A and 1B (CPT1A, CPT1B) are rate limiting FAO enzymes responsible for fatty acid transport from the cytoplasm through the outer mitochondrial membrane. CPT1A gene expression was downregulated in hiBMEC as compared to hpBMEC (Log_2_FC = −5.83), while CPT1B gene expression was upregulated in hiBMEC (Log_2_FC = 8.44). RNA expression of CPT2, which is responsible for fatty acid transport through the inner mitochondrial membrane, was upregulated in hiBMEC as compared to hpBMEC (Log_2_FC = 0.96). Protein levels also largely followed the RNA expression (Fig. 5B, C), although CPT1B protein levels were lower while RNA expression was higher in hiBMEC as compared to hpBMEC (Log_2_FC = −1.06, p = 0.0019). Despite these changes in TCA cycle and FAO enzymes, isotopomer analysis of cells labeled with U-^13^C_6_-glucose revealed no significant differences in the glucose labeled fraction of TCA metabolites (Fig. 5D).

### hpBMEC and hiBMEC metabolism responded similarly to extracellular stimuli

**Fig. 6:**
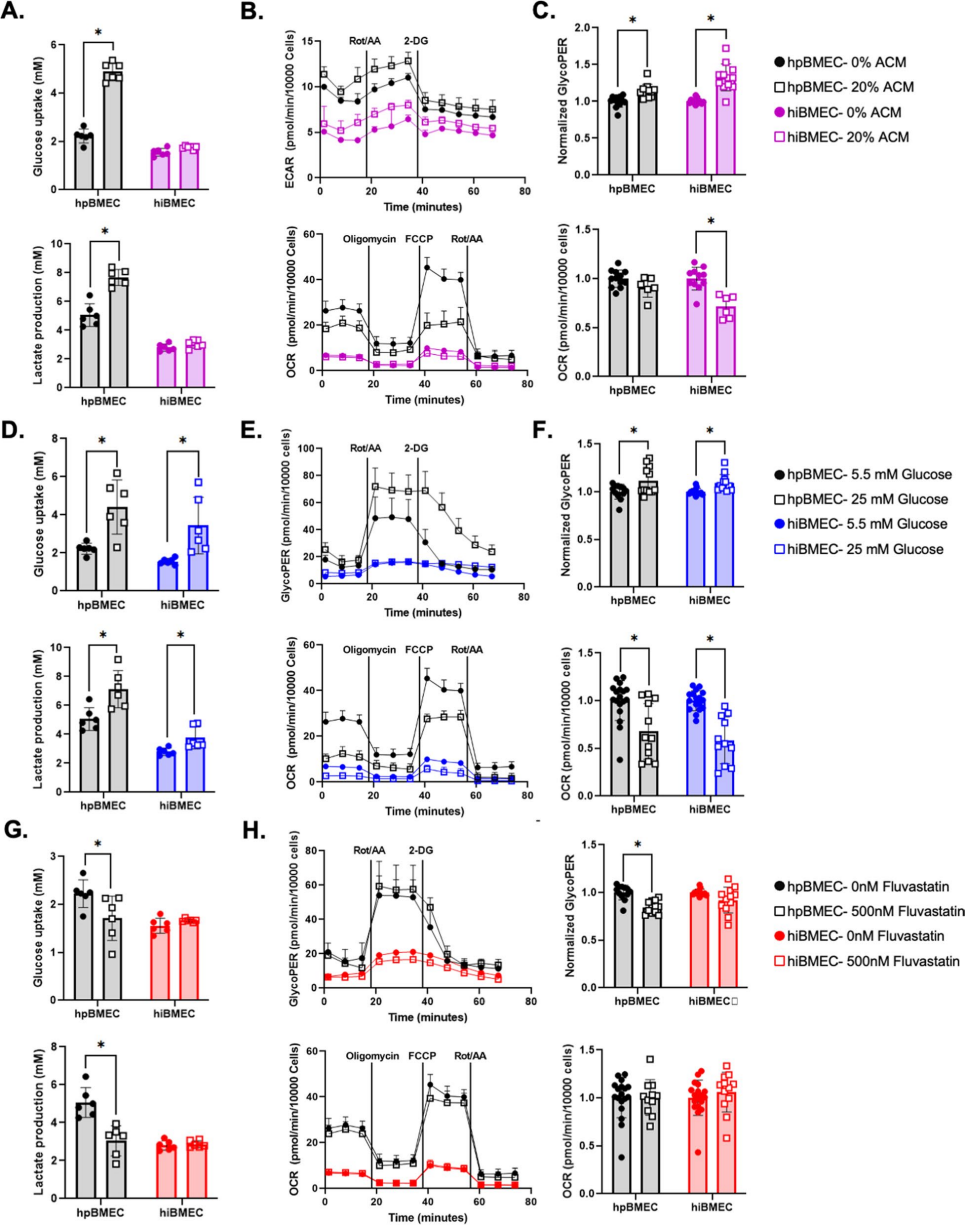
hpBMEC and hiBMEC had similar glycolytic responses to ACM and high glucose, but not fluvastatin. Glucose uptake and lactate production of hpBMEC compared to hiBMEC following a 24-h treatment with **A** 0 or 20% ACM, **D** 5.5 mM or 25 mM glucose, or **G** 0 nM or 500 nM fluvastatin, measured by YSI. n = 6 biological replicates. Seahorse Glycolytic Rate Assay and Mito Stress Test, with analysis of basal GlycoPER and OCR, of hpBMEC compared to hiBMEC following a 24-h treatment with **B**, **C** 0 or 20% ACM, **E**, **F** 5.5 mM or 25 mM glucose, or (H-I) 0 nM or 500 nM fluvastatin. n = 15 biological replicates. *p < 0.05 using a Mann–Whitney test

Finally, we determined if hpBMEC and hiBMEC had similar glucose metabolic responses to external stimuli. Cells were treated for 24 h with 20% astrocyte conditioned media (ACM), high glucose media (25 mM), or 500 nM fluvastatin, after which metabolism was measured with YSI and Seahorse assays. hpBMEC treated with 20% ACM increased glucose uptake (2.22 mM vs. 4.89 mM; p < 0.0001) and lactate production (7.67 mM vs. 5.05 mM; p < 0.0001), but the hiBMEC did not (Fig. 6A). ACM increased GlycoPER in both hpBMEC (14%; p = 0.0065) and hiBMEC (33%; p < 0.0001), while ACM decreased OCR in the hiBMEC only (29%; p < 0.0001; Fig. 6B-C). High glucose increased glucose uptake (2.22 mM vs. 4.42 mM; p = 0.0032) and lactate production (7.13 mM vs. 5.05 mM; p = 0.0009) in both hpBMEC and hiBMEC (Fig. 6D). High glucose also significantly increased GlycoPER (11%, p < 0.0001 in hpBMEC; 9%, p = 0.0001 in hiBMEC) and decreased OCR (32%, p = 0.0003) in hpBMEC; 42%, p < 0.0001 in hiBMEC; Fig. 6E). Finally, we treated BMEC with a statin, since statins have been shown to impair cellular glucose uptake in addition to inhibiting HMG-CoA reductase [42]. Fluvastatin decreased glucose uptake (2.22 mM vs. 1.71 mM; p = 0.0104) and lactate production (5.05 mM vs. 3.05 mM; p < 0.0001) in hpBMEC but not in hiBMEC, and GlycoPER similarly decreased in hpBMEC (16%; p < 0.0001) but not in hiBMEC. OCR was unaffected in both cell types by fluvastatin treatment.

## Discussion

Cerebral glucose metabolic dysfunction is associated with Alzheimer’s [43], Parkinson’s [44], and motor neuron diseases [45]. While hpBMEC are used to model BMEC glucose metabolism [22], they are expensive and have limited availability. We therefore compared glucose metabolism in hiBMEC, a renewable BMEC source, and hpBMEC to determine if hiBMEC can be used to model BBB glucose metabolism. We now show that hpBMEC have higher overall glycolytic and oxidative metabolism than hiBMEC, that the metabolomes of the two cell types are distinct, and that the cells differently express glycolytic, TCA cycle, and FAO enzymes. However, the metabolomic differences lie primarily in the acylcarnitine pathway. The two cell types have similar glucose:lactate ratios, and the glucose labeled fractions of glycolytic and TCA cycle metabolites are similar between the two cell types. We thus propose that hiBMEC have similar glucose metabolism as hpBMEC.

hpBMEC had higher glycolytic and oxidative metabolic rates compared to hiBMEC, which is interesting given that stem cells generally have high metabolic rates [46]. Recent work by Williams et al. Further suggests that hiBMEC glycolysis decreased with extended culture, which would have increased metabolic differences between the two cell types [47]. The differences between hpBMEC and hiBMEC glycolytic rates could relate to changes in GLUT1 localization, which was diffuse throughout hpBMEC and localized to cell–cell junctions in hiBMEC, or to changes in metabolic enzymes [48, 49]. The higher glycolytic flux in hpBMEC could also impact the way that these cells interact metabolically with other NVU cells. Thus, glucose metabolism together with transport should be compared between hpBMEC and hiBMEC in NVU models that also contain pericytes and astrocytes.

Despite the difference in overall metabolic flux, the two cell types had similar lactate-to-glucose ratios and glucose-labeled fractions. This indicates that glucose taken up by the cells is similarly consumed in glycolysis, its side branch pathways (e.g., polyol and hexosamine biosynthesis), and the TCA cycle. In preliminary experiments, we further determined that immortalized HCMEC/D3 BMEC have similar glycolytic and oxidative metabolism to hpBMEC (data not shown), which agrees with published studies [22]. All three cell types produced more ATP via glycolysis than via the TCA cycle, confirming what had previously been reported only in hpBMEC [22]. Peripheral endothelial cells have consistently been shown to primarily use glycolysis for ATP production, and glycolysis is essential to endothelial cell migration and angiogenesis [50]. The reliance of all these cells on glycolysis for ATP production indicates that they likely share fundamental endothelial metabolic characteristics.

Through hypergeometric analysis, we identified a significant difference in the acylcarnitine pathway between hpBMEC and hiBMEC, with five short-chain acylcarnitines significantly elevated in hiBMEC. Acylcarnitines are synthesized when an acyl group is transferred from a fatty acid or an acyl-CoA to free L-carnitine in reactions catalyzed by CPT1A, CPT1B, carnitine acetyltransferase (CrAT), and carnitine octanoyltransferase (CrOT) [51]. Elevated acylcarnitines in hiBMEC may be a remnant metabolic signature of hiBMEC differentiation, since acylcarnitines promoted cardiac and neuronal differentiation [52, 53]. The acylcarnitine with the highest VIP score, acetylcarnitine, is postulated to reduce mitochondrial acetyl-CoA, which could then promote glucose oxidation. Overall, the acylcarnitine changes, and the fact that several of the enzymes that catalyze acylcarnitine formation (CPT1A and CPT1B) were downregulated in hiBMEC as compared to hpBMEC, suggest that there are differences in mitochondrial FAO between hpBMEC and hiBMEC. Additional studies into the source and impact of elevated acylcarnitines in hiBMEC are needed.

Despite differences in gene expression and protein levels of rate-limiting enzymes for glycolysis (HK, PFK/PFKFB3, PKM), pentose phosphate pathway (PPP; G6PDH), hexosamine biosynthetic pathway (HBP; GFAT), and the TCA cycle (IDH) between hpBMEC and hiBMEC, the glucose-labeled metabolite fractions were similar between the two cell types. It is possible that the different isoforms have conflicting effects, or that the enzymes are regulated at a post-translational level (e.g., by phosphorylation). Based on our RNA-sequencing data, HK1 is the predominant hexokinase isoform in hpBMEC and expressed almost equally with HK2 in hiBMEC. Higher HK1 expression is consistent with previously published data showing HK1 is the predominant isoform in brain homogenates [54]. iPSC, however, have elevated HK2 [55], so the higher HK2 expression in hiBMEC may be an artifact from incomplete differentiation. Indeed, prolonged hiBMEC culture (9 days vs. 2 days past subculture) significantly reduced HK2 [47].

PFKFB3 differences in hpBMEC and hiBMEC were in opposite directions for gene expression (lower in hiBMEC) and protein (higher in hiBMEC). PFKFB3 contains AU-rich elements (ARE), which bind to tristetraproline, a protein that enhances PFKFB3 degradation [56]. Our RNA sequencing data indicate that hiBMEC have lower tristetraproline expression compared to hpBMEC, which may decrease PFKFB3 degradation and cause the higher protein levels. Extended BMEC culture decreased PFKFB3 protein [47], suggesting that the lower gene expression does lower PFKFB3 protein over time.

hpBMEC and hiBMEC glucose metabolism increased in similar although not identical ways in response to ACM. ACM was previously shown to increase cerebral endothelial cell glucose uptake by 23% [57], which is similar to what we observed in the increased GlycoPER of both hpBMEC and hiBMEC. While the GlycoPER data agree with the YSI data for the hpBMEC, the hiBMEC did not shown increased glucose uptake by YSI, which could relate to a difference in sensitivity or timescale between the assays. Only hiBMEC had a statistically significant decrease in OCR with ACM. In prior studies, endothelial cells incubated with ACM increased transforming growth factor-β (TGFβ), a cytokine that significantly decreases OCR [58, 59]. Therefore, it is possible that ACM increases hiBMEC TGFβ to decrease OCR.

Hyperglycemia increased glucose uptake, lactate production, and GlycoPER while decreasing OCR in both hpBMEC and hiBMEC. Acute endothelial cell exposure to high glucose increases glycolysis [60] and decreases mitochondrial respiration [61]. Hyperglycemia can lead to enhanced BBB permeability [62], reactive oxygen species production, and apoptosis [63]. These data suggest that hpBMEC and hiBMEC respond similarly to acute hyperglycemia; however, further tests are needed to assess metabolic changes during prolonged hyperglycemia.

In contrast, only hpBMEC but not hiBMEC decreased glycolysis in response to statin treatment. Statins decrease cholesterol production, which could prove therapeutic in restoring BBB function in neurodegenerative diseases associated with dysregulation of cholesterol homeostasis, including Alzheimer’s, Parkinson’s, and Niemann-Pick type C diseases [64]. Our hpBMEC data agree with studies in which statins decrease glycolysis, glycolytic capacity, mitochondrial respiration, and ATP production [65]. Statins may not have decreased glycolysis in hiBMEC due to elevated HMG CoA reductase expression in hiBMEC (Log_2_FC = 2.26). Thus, the physiologic statin dose that we used might have been too low to induce a measurable effect in hiBMEC.

## Limitations

Although our data show similarities between hpBMEC and hiBMEC glucose metabolism, our study is not without limitations. We used hpBMEC as the more physiologically relevant cell source; however, hpBMEC cultured in vitro also may not accurately model in vivo BMEC metabolism [66]. We used hpBMEC from only one donor and hiBMEC derived from only one iPSC line. As a preliminary exploration, we performed RNA-sequencing on hiBMEC derived from DF19-11-T iPSC. We measured metabolic enzyme Log_2_FC values that followed the same trend as the IMR90 hiBMEC (data not shown). We also differentiated the iPSC with a single protocol [23]. Thus, our study does not account for donor-to-donor variability or other differentiation strategies, which may change metabolism [67]. Additionally, all cells were cultured in static culture and on stiff substrates, both of which are known to impact endothelial metabolism [68–70]. Finally, we tested only a single statin dose based on blood statin concentrations from clinical pharmacokinetics studies [71]. However, this dose may not have been the most relevant for BMEC [72].

## Conclusions

While there are essential differences in hpBMEC and hiBMEC glycolytic and oxidative metabolism, metabolomes, and metabolic enzyme levels, this study suggests that hpBMEC and hiBMEC have similar glucose metabolism and respond similarly to extracellular stimuli known to impact glycolytic activity.

## Supplementary Information

**Additional file 1: Figure S1**. hpBMEC have elevated glycolytic and mitochondrial energy production compared to hiBMEC. (A) Seahorse ATP-rate assay calculations of glycolytic and mitochondrial ATP production used to quantify (B) glycolytic:mitochondrial ATP production ratio. (C) Glycolytic capacity quantified as the change between basal ECAR and ECAR following oligomycin treatment. (D). Maximal respiration quantified as the difference between FCCP-stimulated OCR and rotenone/antimycin-A inhibited OCR. (E) Proton leak defined as the difference between oligomycin-inhibited OCR and rotenone/antimycin-A inhibited OCR. (F). Spare capacity calculated as the difference between basal OCR and FCCP-stimulated OCR. (G). The amount of mitochondrial respiration linked to ATP production calculated as the difference between basal OCR and oligomycin-inhibited OCR. n = 12 *p<0.05 with a Mann-Whitney test. **Table S1**. Hypergeometric analysis of metabolites from mass spectrometry. **Table S2**. Glycolytic enzymes and metabolites. **Table S3.** Fatty acid oxidation and tricarboxylic acid cycle enzymes and metabolites.

## Data Availability

hiBMEC datasets generated and analyzed during the current study are available in the GEO repository under GSE211648 and were compared to hpBMEC under GSE138025 [24].
