# Supplementary material for: Induced pluripotent stem cell-derived cells model brain microvascular endothelial cell glucose metabolism
Source: Fluids Barriers CNS. 2022 Dec 9;19:98. doi: 10.1186/s12987-022-00395-z (PMC9733016; doi:10.1186/s12987-022-00395-z)
Supplement: Supplementary file 1 — Additional file 1: Figure S1. hpBMEC have elevated glycolytic and mitochondrial energy production compared to hiBMEC. (A) Seahorse ATP-rate assay calculations of glycolytic and mitochondrial ATP production used to quantify (B) glycolytic:mitochondrial ATP production ratio. (C) Glycolytic capacity quantified as the change between basal ECAR and ECAR following oligomycin treatment. (D). Maximal respiration quantified as the difference between FCCP-stimulated OCR and rotenone/antimycin-A inhibited OCR. (E) Proton leak defined as the difference between oligomycin-inhibited OCR and rotenone/antimycin-A inhibited OCR. (F). Spare capacity calculated as the difference between basal OCR and FCCP-stimulated OCR. (G). The amount of mitochondrial respiration linked to ATP production calculated as the difference between basal OCR and oligomycin-inhibited OCR. n = 12 *p<0.05 with a Mann-Whitney test. Table S1. Hypergeometric analysis of metabolites from mass spectrometry. Table S2. Glycolytic enzymes and metabolites. Table S3. Fatty acid oxidation and tricarboxylic acid cycle enzymes and metabolites. [file 12987_2022_395_MOESM1_ESM.docx]

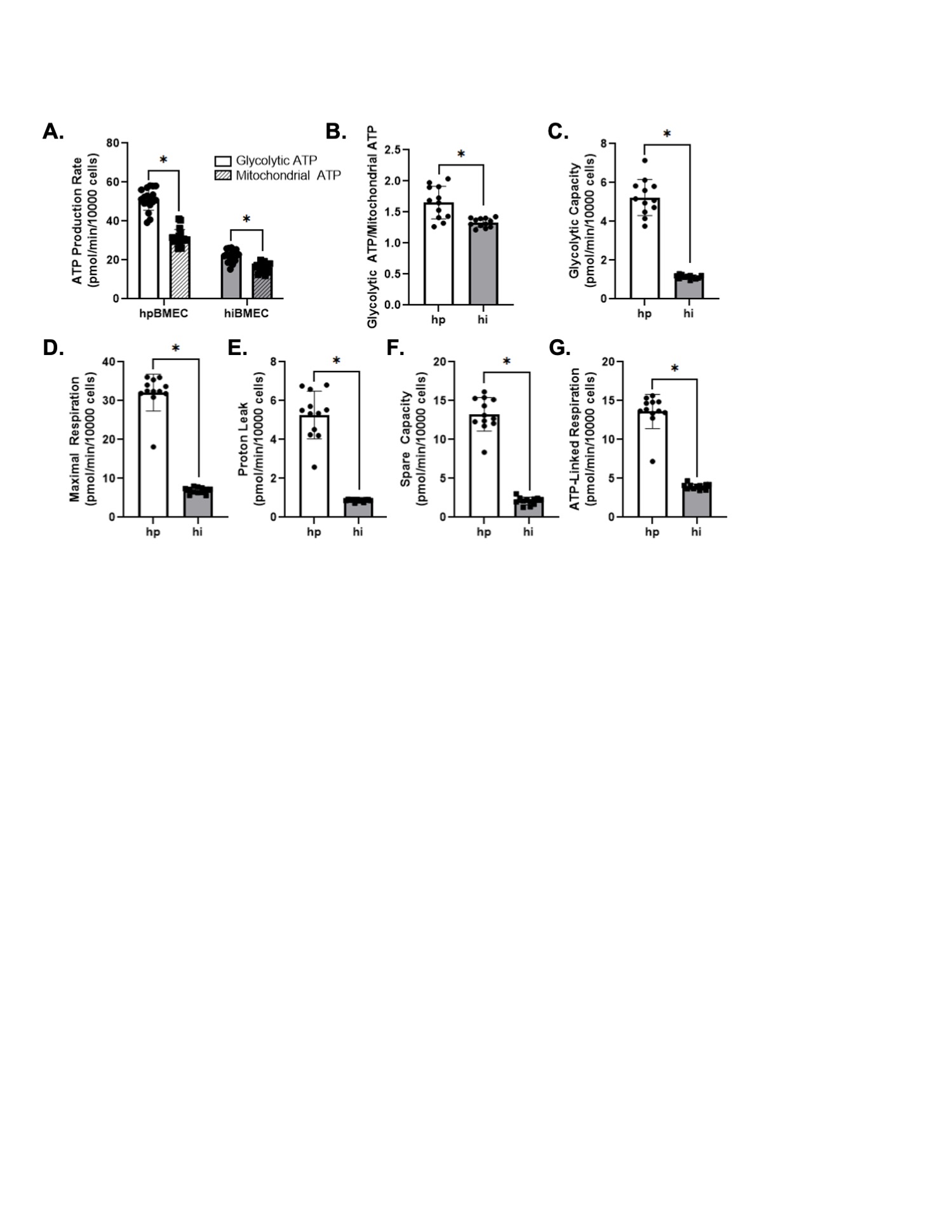


**Additional file Figure 1. hpBMEC have elevated glycolytic and mitochondrial energy production compared to hiBMEC.** (A) Seahorse ATP-rate assay calculations of glycolytic and mitochondrial ATP production used to quantify (B) glycolytic:mitochondrial ATP production ratio. (C) Glycolytic capacity quantified as the change between basal ECAR and ECAR following oligomycin treatment. (D). Maximal respiration quantified as the difference between FCCP-stimulated OCR and rotenone/antimycin-A inhibited OCR. (E) Proton leak defined as the difference between oligomycin-inhibited OCR and rotenone/antimycin-A inhibited OCR. (F). Spare capacity calculated as the difference between basal OCR and FCCP-stimulated OCR. (G). The amount of mitochondrial respiration linked to ATP production calculated as the difference between basal OCR and oligomycin-inhibited OCR. n = 12 *p<0.05 with a Mann-Whitney test.

**Additional file Table 1. Hypergeometric analysis of metabolites from mass spectrometry.**

| Reaction ID | # of differentially expressed metabolites | # of total metabolites in pathway | % of different metabolites | p-value |
| --- | --- | --- | --- | --- |
| Amino acids | 2 | 16 | 0.13 | 0.28 |
| Arginine and proline metabolism | 1 | 7 | 0.14 | 0.40 |
| Carnitine and fatty acid metabolism | 5 | 11 | 0.45 | 0.01 |
| Gamma-glutamyls | 1 | 5 | 0.20 | 0.41 |
| Glycerophospholipid biosynthesis | 2 | 6 | 0.33 | 0.19 |
| Glycolysis | 1 | 6 | 0.17 | 0.41 |
| Nucleotides | 4 | 26 | 0.15 | 0.23 |
| Other sugars | 1 | 4 | 0.25 | 0.39 |
| Phosphates | 1 | 1 | 1.00 | 0.16 |
| Serine biosynthesis and one-carbon metabolism | 1 | 4 | 0.25 | 0.39 |
| Sulfur metabolism | 3 | 8 | 0.38 | 0.09 |
| Urea cycle | 2 | 2 | 1.00 | 0.02 |

**Additional file Table 2. Glycolytic enzymes and metabolites.**

| Abbreviation | Full name |
| --- | --- |
| GLUT1 | Glucose transporter 1 |
| HK1 | Hexokinase 1 |
| HK2 | Hexokinase 2 |
| G6PDH | Glucose-6-phosphate-dehydrogenase |
| GPI | Glucose-6-phosphate isomerase |
| PFKFB3 | 6-phosphofructo-2-kinase/fructose-2,6-biophsphatase 3 |
| PFKL | Phosphofructokinase, liver type |
| PFKM | Phosphofructokinase, muscle type |
| PFKP | Phosphofructokinase, platelet type |
| ALDOA | Aldolase, Fructose-biphosphate A |
| GAPDH | Glyceraldehyde-3-phosphate dehydrogenase |
| PGK | Phosphoglycerate kinase |
| PGM | Phosphoglycerate mutase |
| ENO1 | Alpha-enolase |
| PKM | Pyruvate kinase |
| LDHA | Lactate dehydrogenase A |
| LDHB | Lactate dehydrogenase B |
| GLC | Glucose |
| G6P | Glucose-6 phosphate |
| G1P | Glucose 1-phosphate |
| F6P | Fructose-6 phosphate |
| PYR | Pyruvate |
| LAC | Lactate |
| R5P | Ribose 5-phosphate |
| UDPG | UDP-glucose |
| UACGAM | UDP-N-acetyl-D-glucosamine |

**Additional file Table 3. Fatty acid oxidation and tricarboxylic acid cycle enzymes and metabolites.**

| Abbreviation | Full name |
| --- | --- |
| FABP3 | Fatty acid binding protein 3 |
| FABP4 | Fatty acid binding protein 4 |
| FABP5 | Fatty acid binding protein 5 |
| CPT1A | Carnitine palmitoyltransferase 1A |
| CPT1B | Carnitine palmitoyltransferase 1B |
| CPT1C | Carnitine palmitoyltransferase 1C |
| CPT2 | Carnitine palmitoyltransferase 2 |
| ACADS | Acyl-CoA dehydrogenase short chain |
| ACADM | Acyl-CoA dehydrogenase medium chain |
| ACADVL | Acyl-CoA dehydrogenase very long chain |
| HADHA | Hydroxyacyl-CoA dehydrogenase trifunctional multienzyme complex subunit alpha |
| HADHB | Hydroxyacyl-CoA dehydrogenase trifunctional multienzyme complex subunit beta |
| ACAA1 | Acetyl-CoA acyltransferase 1 |
| ACAA2 | Acetyl-CoA acyltransferase 2 |
| ACLY | ATP citrate lyase |
| CS | Citrate synthase |
| ACO1 | Aconitase 1 |
| ACO2 | Aconitase 2 |
| IDH1 | Isocitrate dehydrogenase 1 |
| IDH2 | Isocitrate dehydrogenase 2 |
| IDH3A | Isocitrate dehydrogenase 3 [NAD] subunit alpha |
| IDH3B | Isocitrate dehydrogenase 3 [NAD] subunit beta |
| IDH3G | Isocitrate dehydrogenase 3 [NAD] subunit gamma |
| OGDH | Oxoglutarate dehydrogenase |
| DLD | Dihydrolipoamide dehydrogenase |
| DLST | Dihydrolipoamide S-succinyltransferase |
| SUCLA2 | Succinate-CoA ligase ADP-forming subunit beta |
| SUCLG1 | Succinate-CoA ligase GDP/ADP-forming subunit alpha |
| SUCLG2 | Succinate-CoA ligase GDP/ADP-forming subunit beta |
| SDHA | Succinate dehydrogenase complex flavoprotein subunit A |
| SDHB | Succinate dehydrogenase complex flavoprotein subunit B |
| SDHC | Succinate dehydrogenase complex flavoprotein subunit C |
| SDHD | Succinate dehydrogenase complex flavoprotein subunit D |
| FH | Fumarate hydratase |
| MDH1 | Malate dehydrogenase 1 |
| MDH2 | Malate dehydrogenase 2 |
| CIT | Citrate |
| GLT | Glutamate |
| SUCC | Succinate |
| MAL | Malate |
| ASP | Aspartate |
